# 6-Gingerol Activates PI3K/Akt and Inhibits Apoptosis to Attenuate Myocardial Ischemia/Reperfusion Injury

**DOI:** 10.1155/2018/9024034

**Published:** 2018-03-20

**Authors:** Xiangwei Lv, Tongtong Xu, Qi Wu, Yao Zhou, Guidong Huang, Yongnan Xu, Guoqiang Zhong

**Affiliations:** ^1^Department of Cardiology, First Affiliated Hospital of Guangxi Medical University, Nanning, Guangxi Zhuang Autonomous Region 530021, China; ^2^Department of Integrated Traditional Chinese and Western Medicine, First Affiliated Hospital of Guilin Medical University, Guilin, Guangxi Zhuang Autonomous Region 541001, China; ^3^Department of Respiratory, Affiliated Hospital of Nanjing University of Chinese Medicine, Nanjing, Jiangsu 210029, China; ^4^Department of Pharmacy, First Affiliated Hospital of Guilin Medical University, Guilin, Guangxi Zhuang Autonomous Region 541001, China; ^5^Department of Infectious Diseases, First Affiliated Hospital of Guilin Medical University, Guilin, Guangxi Zhuang Autonomous Region 541001, China

## Abstract

6-Gingerol (6-G) is known to alleviate myocardial ischemia/reperfusion injury. However, the underlying molecular mechanisms of 6-G myocardial protection are not known. In this study, the protective effect of 6-G on ischemia/reperfusion (I/R) damage and whether such a mechanism was related to apoptosis inhibition and activation of phosphoinositide 3-kinases (PI3K)/serine/threonine kinase (Akt) signaling pathway were investigated. Rats were subjected to I/R in the presence or absence of 6-G and the changes of cardiac function, infarct size and histopathological changes, and the levels of cardiac troponin T, creatine kinase-MB, and myocardial apoptosis were examined. The expression of caspase-3, PI3K, p-Akt, and Akt was also determined. We found that 6-G (6 mg/kg) pretreatment significantly improved heart function and ameliorated infarct size and histopathological changes and cardiac troponin T and creatine kinase-MB levels induced by I/R. Moreover, pretreatment with 6-G significantly inhibited myocardial apoptosis and caspase-3 activation induced by I/R. 6-G also upregulated expression of PI3K, p-Akt, and Akt in myocardial tissues. Taken together, these findings suggest that 6-G inhibits apoptosis and activates PI3K/Akt signaling in response to myocardial I/R injury as a possible mechanism to attenuate I/R-induced injury in heart. These results might be important for developing novel strategies for preventing myocardial I/R injury.

## 1. Introduction

Acute myocardial infarction (AMI) has become a global health problem [1]. In 2013, more than 930,000 rural people and 720,000 urban people in China died of AMI, and the number is expected to rise in the next decade [2]. Percutaneous coronary intervention (PCI) and thrombolytic therapy can recover myocardial blood supply in timely manner. These are the most effective treatments for saving the endangered myocardium, reducing the infarct size, maintaining cardiac function, and alleviating ventricular remodeling after infarction [3]. Myocardial ischemia/reperfusion injury (MIRI) causes damage to the function, metabolism, and structure of the heart. It also induces myocardial apoptosis, inflammatory response, and oxidative stress resulting in severe arrhythmia, cardiac failure, and even sudden cardiac death [4]. Although patients with AMI may undergo timely reperfusion therapy, the mortality of MIRI is still 10% [5]. Therefore, there is an urgent need for new drugs that could prevent and treat MIRI.

Apoptosis is an active and orderly cell death process regulated by genes and involves a series of enzymes and is currently the recognized mechanism of MIRI [6]. Studies have demonstrated the occurrence of apoptosis in early stages of myocardial infarction which increases significantly during reperfusion and these are important ways for myocardial cell death in AMI and MIRI [7, 8]. Therefore, inhibition of cardiomyocyte apoptosis may improve cardiac function and alleviate MIRI which is of clinical significance for treating myocardial infarction.

Phosphoinositide 3-kinases/serine/threonine kinase (PI3K/Akt) signaling pathway is an important cell survival mechanism. It has important biological functions in cell survival, apoptosis, and proliferation [9, 10]. Fujio et al. [11] demonstrated that PI3K/Akt signaling pathway serves as an endogenous negative-feedback regulation or compensatory mechanism that could inhibit harmful stimuli-induced apoptotic events. Negoro et al. [12] reported that PI3K/Akt pathway transduces signals that inhibit apoptosis in cardiomyocyte in response to MIRI. Other studies have demonstrated that PI3K/Akt signaling pathway alleviates MIRI by inhibiting cardiomyocyte apoptosis induced by oxidative stress [13–15].

Recent epidemiological studies have shown that extracts from herbs, vegetables, fruits, and spices can reduce the risk of cardiovascular diseases [16, 17]. 6-Gingerol (6-G) is a phenolic substance extracted from ginger. It is a major component of gingerols and has significant antiapoptotic, antioxidative, and anti-inflammatory properties [18, 19]. Sampath et al. [20] showed that 6-G prevents atherosclerosis by inhibiting oxidative stress-induced apoptosis. El-Bakly et al. [21] reported that 6-G has a significant effect in myocardial protection by inhibiting apoptosis through antioxidation and alleviation of doxorubicin-induced myocardial injury.

A previous study found that 6-G had antioxidative effects, inhibiting myocardial cell apoptosis and alleviating MIRI [22]. However the mechanisms of how 6-G prevents apoptosis and MIRI remain unexplored. This study tested the hypothesis that 6-G may activate PI3K/Akt pathway as a possible mechanism of inhibition of apoptosis induced by MIRI.

## 2. Materials and Methods

### 2.1. Drugs and Reagents

6-G (purity, ≥95%) and 2,3,5-triphenyltetrazolium chloride (TTC) were acquired from Sigma Chemical Co. (MO, USA). Cardiac troponin T (cTnT), creatine kinase-MB (CK-MB), caspase-3, PI3K, and Akt enzyme-linked immunosorbent assay (ELISA) kits were obtained from the Cusabio Biotech Co. (MD, USA). The terminal deoxynucleotidyl transferase-mediated dUTP-biotin nick end-labeling (TUNEL) apoptosis detection kit was obtained from the Roche Diagnostics (Mannheim, Germany). Caspase-3, PI3K, p-Akt, Akt, and *β*-tubulin antibodies were purchased from Cell Signaling Technology, Inc. (MA, USA); BCA protein quantitative assay kit and secondary antibodies were procured from Beyotime Biotechnology, Inc. (Changsha, China).

### 2.2. Animals and Establishment of Rat Myocardial I/R Mode

Sprague-Dawley male rats (weighing 200–280 g) were supplied by the Laboratory Animal Center (SCXK [Gui] 2014-0003) of Guangxi Medical University. All of the procedures and protocols used in our experiments were approved by the Ethics Committee for the Experimental Use of Animals in Guangxi Medical University. Rats were housed in cages under standard experimental conditions, at 20°C–25°C with the humidity of 50%–60% in 12-h light/dark cycle. Animals were allowed free access to standard tap water and rodent chow.

Thirty-two rats were randomized into four groups with sample number of eight for each group. (1) Sham group: rats in this group underwent surgery, but the left anterior descending coronary artery (LAD) was not ligated; (2) 6-G (6 mg/kg) group: the tail vein was injected with 6-G in this group 30 min before surgery: LAD was not ligated; (3) I/R group: the LAD was ligated for 30 min followed by reperfusion for 2 h; (4) 6-G (6 mg/kg) + I/R group: the tail vein was injected 30 min before surgery and LAD was ligated for 30 min. Finally, reperfusion was performed for 2 h.

Rats were intraperitoneally anesthetized using 4% chloral hydrate (10 mL/kg), with dosage maintained at 0.5 mL/kg for the study. After tracheal intubation, ventilation was introduced using a small-animal ventilator at the respiratory rate of 80–90 cycles/min, the expiratory-to-inspiratory ratio of 2 : 1, and the tidal volume of 8–10 mL/kg. Then, a left thoracotomy was done in the 4th intercostal space. Subsequently, to expose the heart, the LAD was ligated with 6-0 thread. The ligation thread was loosened after a 30 min ischemia, and then a 2 h reperfusion was performed. Limb-lead electrocardiographic electrodes were inserted into the limbs of the rats and electrocardiographic changes were monitored. Elevation of electrocardiogram (ECG) ST-segment and gray-white or swollen myocardium in the blood supply area of ligated blood vessels indicated that the model was successfully established.

### 2.3. Heart Function Measurement

The invasive hemodynamics evaluation method was used to evaluate the cardiac dysfunction caused by ischemia reperfusion. One end of a microtubing was intubated into the rat left ventricle through its right common carotid artery, while the other end was connected to MS400 biological signal analysis system (Longfeida Technology Co., Ltd., Shandong, China). After reperfusion, left ventricular ejection fraction (EF), left ventricular fractional shortening (FS), left ventricular end systolic diameter (LVESD), left ventricular end diastolic diameter (LVEDd), and maximum rise/down velocity of left intraventricular pressure (±*dp*/*dt*_max_) were recorded. Data used for statistical analysis was the mean value of three cardiac cycles.

### 2.4. 2,3,5-Triphenyltetrazolium Chloride, Hematoxylin, and Eosin Staining Method for Infarct Size and Histopathological Examination of Myocardial Tissues

The infarct size of heart was determined by 1% TTC staining method following the manufacturer's instructions. Briefly, at completion of reperfusion, the hearts samples were collected and placed at −20°C for 20 min and then sliced into approximately 2 mm thick specimens which were next incubated for 20 min in 1% TTC at 37°C. White represents infarct area and red indicates noninfarct area, while the infarct size was measured by Image-Pro Plus 6.0 software. The myocardial tissue was fixed in 4% paraformaldehyde, embedded in paraffin, and subsequently stained with hematoxylin and eosin (H&E). Myocardial injury was observed using an optical microscope (CKX41, Olympus, Tokyo, Japan). MIRI was scored according to the following morphological criteria, as defined as described [23]. Score 0: zero damage; Score 1: mild with interstitial edema and local necrosis; Score 2: moderate with extensive myocardial cell swelling and local necrosis; Score 3: severe with contraction band, capillary compression necrosis, and inflammatory cell infiltration; Score 4: highly severe with diffuse necrosis with contraction bands, compressed capillaries, and hemorrhage.

### 2.5. ELISA for Serum Cardiac Troponin T and Creatine Kinase-MB Levels

At completion of reperfusion, the arterial blood samples were collected in a vacuum tube containing heparin and centrifuged at 3000*g* and 4°C for 15 min. The cTnT and CK-MB levels were determined using ELISA kits following the manufacturer's instructions.

### 2.6. TUNEL Assay for Apoptosis in Cardiomyocyte

TUNEL assays were performed to detect myocardial apoptosis using a commercial kit based on the manufacturer's instructions. The apoptotic cell nuclei was stained green and normal nuclei stained blue. Six fields at a magnification of ×200 were randomly selected from each sample using an optical microscope. The calculation of apoptosis index (AI) was performed as the ratio of TUNEL-positive nuclei to the total number of nuclei stained in each field. Image-Pro Plus 6.0 software was employed in our data analysis.

### 2.7. ELISA for Caspase-3, PI3K, and Akt Expression in Myocardial Tissues

Caspase-3, PI3K, and Akt expression in myocardial homogenates were measured using caspase-3, PI3K, and Akt ELISA kits following the manufacturer's instructions.

### 2.8. Western Blot for Caspase-3, PI3K, p-Akt, and Akt Expression in Myocardial Tissues

Myocardial tissue (100 mg) was placed at the globular site of a homogenizer and cut into small pieces with clean scissors. The tissue was homogenized in RIPA buffer containing phenylmethylsulfonyl fluoride (PMSF) on ice for 30 min. The lysate was then transferred to 1.5 ml tubes and centrifuged at 10000–14000 g for 5–10 min at 4°C. The supernatant was boiled for 5 min and protein concentration was determined with BCA test kit. Proteins were run on sodium dodecyl sulfate polyacrylamide gel electrophoresis (SDS-PAGE) and subsequently transferred onto the polyvinylidene difluoride-plus (PVDF) membrane. Then the PVDF membrane was incubated overnight with caspase-3 (1 : 1000), PI3K (1 : 1000), p-Akt (1 : 1000), Akt (1 : 1000), or *β*-tubulin (1 : 1000). Blots were washed and incubated with secondary antibody (1 : 16000) for 2 h at room temperature. The blots were developed using chemiluminescence system (Amersham Pharmacia). The relative density data was collected and analyzed by Image-Pro Plus 6.0 software.

### 2.9. Statistical Data Analysis

Statistical analysis was carried out using the SPSS statistical package (version 21.0; IBM, NY, USA). All results are shown as mean ± standard deviation. Differences between separate groups were evaluated using one-way analysis of variance. *P* values of < 0.05 are considered as statistically significant.

## 3. Results

### 3.1. The Representative Changes in ECG Were Used to Verify the Success of the I/R Model and the Effects of Pretreatment with 6-G on the ECG Pattern

**Figure 1:**
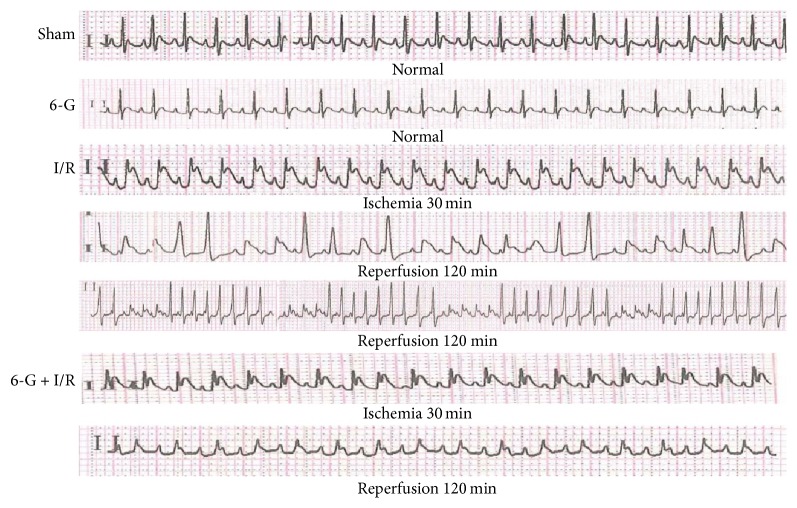
Representative changes in ECG of the I/R model and the effects of pretreatment with 6-G (6 mg/kg) on the ECG pattern.

To confirm the success of the I/R model, we examined the ECG changes. As shown in Figure 1, the ECG pattern was not found to vary in the Sham group and 6-G group. In contrast, remarkable elevation in ST-segment and reperfusion arrhythmia were found in the I/R group, while both were diminished markedly in 6-G pretreatment group.

### 3.2. Pretreatment with 6-G Promotes Recovery of Cardiac Dysfunction in Response to I/R

**Figure 2:**
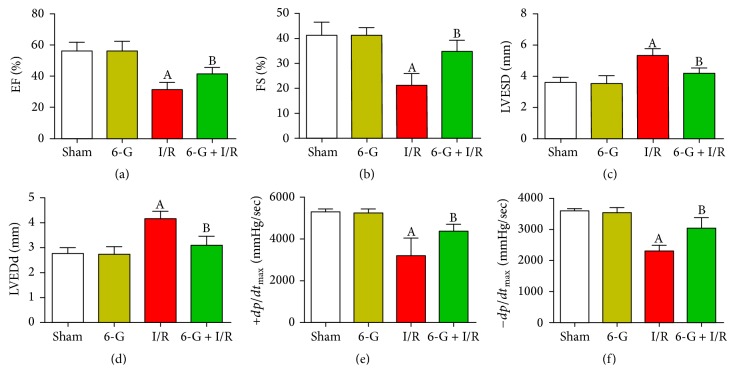
Pretreatment with 6-G (6 mg/kg) improved cardiac dysfunction induced by I/R. (a) EF, left ventricular ejection fraction. (b) FS, left ventricular fractional shortening. (c) LVESD, left ventricular end systolic diameter. (d) LVEDd, left ventricular end diastolic diameter. (e) +*dp*/*dt*_max_, maximum rise velocity of left intraventricular pressure. (f) −*dp*/*dt*_max_, maximum down velocity of left intraventricular pressure. Note that ^A^*P* < 0.05 against the Sham group; ^B^*P* < 0.05 against the I/R group.

To explore the function of 6-G on heart dysfunction recovery after ischemia reperfusion, we followed the changes of EF, FS, LVESD, LVEDd, +*dp*/*dt*_max_, and −*dp*/*dt*_max_ in our experiment. As shown in Figures 2(a) and 2(b), EF and FS in I/R group decreased significantly, while both increased markedly in 6-G pretreatment group (*P* < 0.05). Meanwhile, LVESD and LVEDd in the 6-G pretreatment group decreased significantly (*P* < 0.05, Figures 2(c) and 2(d)), while +*dp*/*dt*_max_ and −*dp*/*dt*_max_ increased markedly (*P* < 0.05, Figures 2(e) and 2(f)), compared to the I/R group.

### 3.3. Pretreatment with 6-G Reverses Infarct Size, Histopathological Changes, and Myocardial Injury Induced by I/R

**Figure 3:**
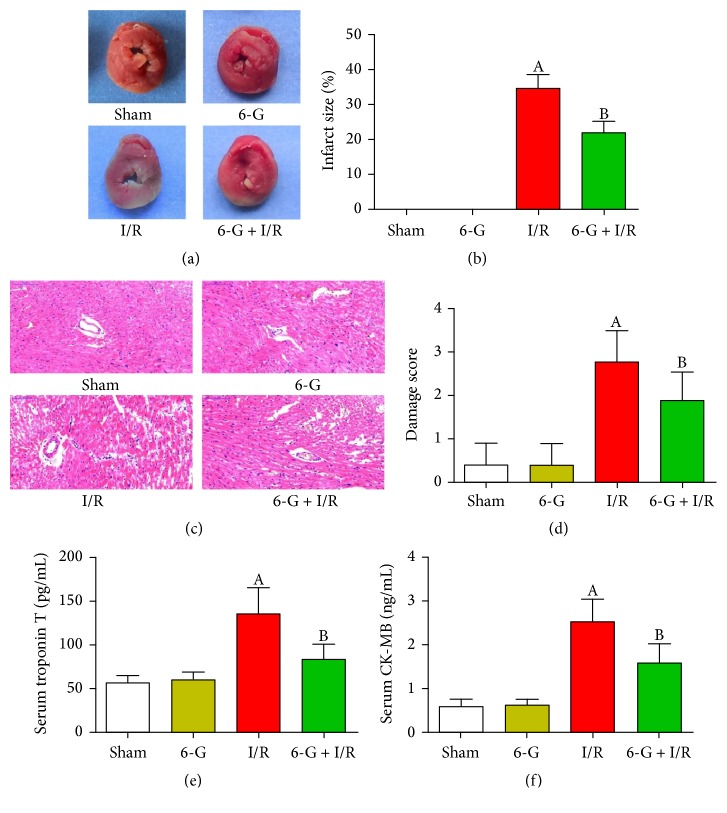
Pretreatment with 6-G (6 mg/kg) reversed infarct size, histopathological changes, and myocardial injury induced by I/R. (a) Representative images of TTC stained samples. White represents infarct area and red indicates noninfarct area. The infarct size can then be measured using Image-Pro Plus 6.0 software. (b) Infarct size score. (c) Representative images of H&E stained samples (×200). (d) Damage score. (e) Serum level of cTnT. (f) Serum level of CK-MB. Note that ^A^*P* < 0.05 against the Sham group; ^B^*P* < 0.05 against the I/R group.

To study the protective effect of 6-G on I/R-induced myocardial injury, we monitored myocardial infarct size and histopathological changes using TTC and H&E staining. Markers of myocardial damage (cTnT and CK-MB) were also determined in the serum. As shown in Figures 3(a) and 3(c), myocardial infarct size was not found in the Sham group and 6-G group; myocardial structure in the Sham group and 6-G group indicated a regular arrangement, normal myocardial fibers, and no necrosis. In contrast, severe myocardial infarct size and damage were found in the I/R group, including ruptured cardiac muscle fibers, inflammatory cell infiltration, and edema. Importantly, myocardial tissues from animals pretreated with 6-G prior to I/R exhibited fewer infarct size and histopathological changes compared to the I/R group. Also, 6-G treated group had significantly lower cTnT and CK-MB levels relative to the I/R group (*P* < 0.05, Figures 3(e) and 3(f)).

### 3.4. Pretreatment with 6-G Inhibits I/R-Induced Apoptosis in Myocardial Tissues

**Figure 4:**
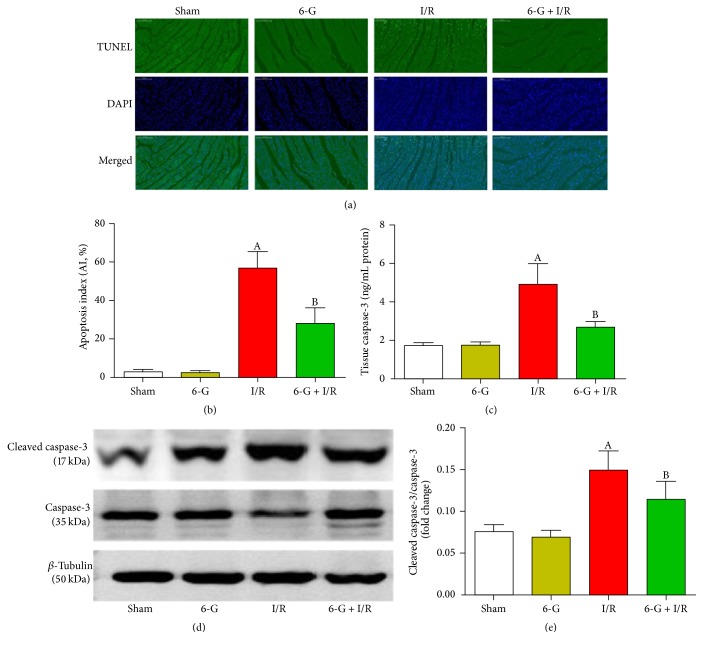
Pretreatment with 6-G (6 mg/kg) inhibited myocardial apoptosis induced by I/R. (a) Representative images from the TUNEL assays (×200) in the myocardial tissues. Green fluorescence represents TUNEL-positive nuclei; blue fluorescence represents nuclei of total cardiac myocytes. (b) Apoptosis index. (c) Caspase-3 expression in myocardial homogenates was evaluated using ELISA. (d) Expression level of cleaved caspase-3 in the myocardial tissues detected using western blot and quantitative analyses (e). Note that ^A^*P* < 0.05 against the Sham group; ^B^*P* < 0.05 against the I/R group.

We confirmed the antiapoptotic effects of 6-G by TUNEL and by the protein expression of caspase-3 and cleaved caspase-3. As shown in Figure 4(a), the number of positive cells of TUNEL in the I/R group increased significantly (*P* < 0.05) compared to the Sham group. In contrast, number of negative cells of TUNEL in 6-G pretreatment group decreased markedly compared to the I/R group (*P* < 0.05). To further confirm the antiapoptotic effects of 6-G, we used ELISA to investigate the expression of caspase-3 in myocardial homogenates and to investigate the expression of cleaved caspase-3 in the cardiac tissues by western blot. As shown in Figure 4(c), the expression of caspase-3 in the I/R group increased significantly (*P* < 0.05) compared to the Sham group, whereas the expression of caspase-3 in the 6-G pretreatment group decreased significantly compared to the I/R group (*P* < 0.05). As shown in Figure 4(e), the expression of cleaved caspase-3 in the I/R group increased significantly (*P* < 0.05) compared to the Sham group, and this effect was overcome also by 6-G pretreatment (*P* < 0.05). All of these results suggest that 6-G reduces cardiac damage induced by ischemia reperfusion via its antiapoptotic effects.

### 3.5. Pretreatment with 6-G Activates PI3K/Akt Signaling Pathway

**Figure 5:**
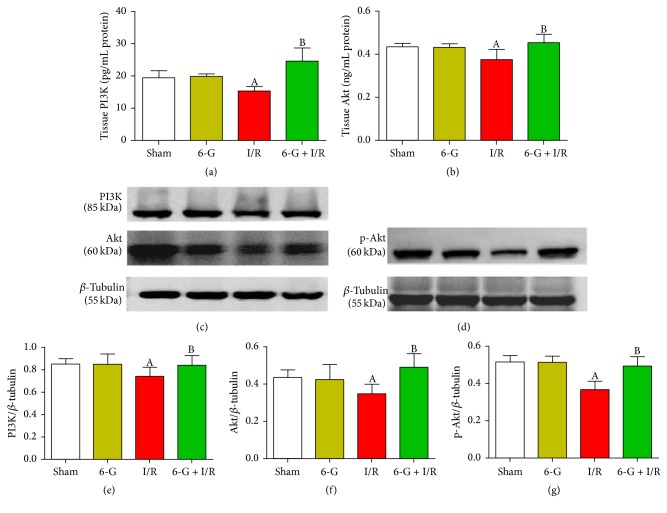
Pretreatment with 6-G (6 mg/kg) activated the PI3K/Akt signaling pathway. ((a) and (b)) PI3K and Akt activities in myocardial homogenates detected by ELISA. ((c) and (d)) The expression level of PI3K, p-Akt, and Akt in the myocardial tissues detected using western blot and quantitative analyses ((e), (f), and (g)). Note that ^A^*P* < 0.05 against the Sham group; ^B^*P* < 0.05 against the I/R group.

We next investigated the expression of PI3K and Akt in myocardial homogenates by ELISA and the expression of PI3K, p-Akt, and Akt in the cardiac tissues by western blot in response to I/R in the presence or absence of 6-G. PI3/Akt pathway has been previously reported to overcome MIRI-induced apoptosis in cardiomyocyte [12]. In myocardial homogenates, as demonstrated in Figures 5(a) and 5(b), PI3K and Akt expression was reduced in I/R group and this effect was reversed in 6-G pretreatment group (*P* < 0.05). A corresponding decrease in protein levels of PI3K, p-Akt, and Akt was also observed in the I/R group compared to the Sham group. Pretreatment with 6-G protected against the inhibitory effect of I/R on these proteins (*P* < 0.05, Figures 5(e), 5(f), and 5(g)).

## 4. Discussion

In the present investigation, we report a previously unknown mechanism of protective effects of 6-G against MIRI. Our findings reveal that 6-G promotes recovery of cardiac dysfunction, alleviates infarct size and pathological changes in myocardial tissues; reduces CK-MB and cTnT levels; and inhibit cardiomyocyte apoptosis induced by I/R. It also upregulates the expression of PI3K/Akt signaling pathway.

MIRI is a complex pathophysiological process implicated in apoptosis, inflammatory response, and oxidative stress [24]. Apoptosis is an important form of cardiomyocyte death in the early stage of MIRI and is crucial in the pathophysiology of MIRI, which further leads to severe complications such as arrhythmia and heart failure [25, 26]. An experimental study found that cardiomyocyte injury of rats was aggravated after I/R, showing typical changes in morphological phenotype of cell apoptosis [27]. Cardiomyocyte apoptosis increased significantly in the reperfusion group than in the permanent ischemia group [28]. A clinical study found that cardiomyocyte apoptosis occurred in patients with acute coronary syndrome before and after PCI [29]. In patients with AMI, DNA strand breaks in cardiomyocyte were observed in the regions adjacent to and further away from the necrotic myocardium. This was further confirmed by DNA gradient gel electrophoresis and apoptotic morphologies showed typical changes [30].

I/R may lead to activation of cascade of apoptotic proteins called caspases. A recent study found that more than 14 kinds of caspases were involved in regulating cell apoptosis. Among these, caspase-3 is the main executor of cell apoptosis which directly causes cell death, intracellular protein dissolution, and degradation of enzymes and DNA [31]. An experimental study showed that melatonin downregulates expression of caspase-3 to alleviate MIRI by activating sirtuin-3 signaling pathway and by increasing the expression of Tom70 [32, 33]. Vanillyl alcohol reduces the activation of caspase-3 to alleviate MIRI by antioxidation [34]. Schisandrin B downregulates expression of caspase-3 to attenuate endoplasmic reticulum stress-induced cardiomyocyte apoptosis via transcription factor 6 and (PKR-) like ER kinase signaling pathways, further alleviating MIRI [35]. These findings suggest that downregulation of expression of caspase-3 may alleviate MIRI. In the present study, we found that 6-G downregulated the caspase-3 expression to inhibit cardiomyocyte apoptosis and alleviated myocardial injury during reperfusion.

Recent studies have demonstrated PI3K/Akt signaling pathway to be important for myocardial protection. It regulates survival and apoptosis of cardiomyocyte by regulating the integrity of cardiomyocyte morphology, protein synthesis, and metabolic function [36, 37]. Various studies have underscored the involvement of PI3/Akt pathway in protecting against MIRI-induced apoptosis. Neural precursor cell expressed developmentally downregulated 4-1 (NEDD4-1) was found to downregulate expression of caspase-3 to protect against cardiomyocyte apoptosis generated through I/R by activating PI3K/Akt signaling pathway [38]. Also, microRNA-214 regulates expression of phosphatase and tensin homolog by activating PI3K/Akt pathway and downregulating caspase-3 expression to alleviate I/R-induced cardiomyocyte apoptosis [39]. Another study reported that Gypenoside inhibits cardiomyocyte apoptosis by activating PI3K/Akt* in vivo* and* in vitro* to protect against MIRI [40]. These results confirm that activating the signaling pathway of PI3K/Akt in the heart may alleviate MIRI.

In the present study, we found that 6-G activated PI3K/Akt signaling pathway as a possible mechanism of apoptosis inhibition in response to MIRI. However, the present study has some limitations. Firstly, the main purpose of our study is the preventive effect of 6-G. We thus focused on evaluating the use of 6-G before PCI or thrombolysis to reduce the subsequent appearance of MIRI. The study of 6-G treatment effect after ligation of LAD will be conducted in a subsequent and separate study. Secondly, the findings in our experiments were based on a vivo animal model, but not from direct clinical situations of ST-segment elevation myocardial infarction (STEMI). Further investigation will be needed to verify our findings in clinical scenario. Thirdly, this study did not use inhibitors of PI3K/Akt signaling pathway such as LY294002 or wortmannin or siRNA approach and these research results are part of a separate investigation of ours. We are currently conducting the PI3K/Akt signaling pathway blockade study. In conclusion, the current study shows that 6-G alleviates MIRI by inhibiting I/R-induced cardiomyocyte apoptosis and that 6-G directly or indirectly activates PI3K/Akt signaling pathway. These experimental results suggest that the application of 6-G may offer a new strategy for preventing MIRI.
